# Electrosynthesis of Electrochromic Polymer Membranes Based on 3,6-Di(2-thienyl)carbazole and Thiophene Derivatives

**DOI:** 10.3390/membranes11020125

**Published:** 2021-02-09

**Authors:** Chung-Wen Kuo, Jui-Cheng Chang, Jeng-Kuei Chang, Sheng-Wei Huang, Pei-Ying Lee, Tzi-Yi Wu

**Affiliations:** 1 Department of Chemical and Materials Engineering, National Kaohsiung University of Science and Technology, Kaohsiung 80778, Taiwan; welly@nkust.edu.tw (C.-W.K.); bill4794@gmail.com (S.-W.H.); 2 Department of Chemical Engineering and Materials Engineering, National Yunlin University of Science and Technology, Yunlin 64002, Taiwan; d700215@gmail.com (J.-C.C.); leepeiying1018@gmail.com (P.-Y.L.); 3 Bachelor Program in Interdisciplinary Studies, National Yunlin University of Science and Technology, Yunlin 64002, Taiwan; 4 Department of Materials Science and Engineering, National Chiao Tung University, No. 1001 University Road, Hsinchu 30010, Taiwan; jkchang@nctu.edu.tw

**Keywords:** electrochromic behavior, spectroelectrochemical property, transmittance, dual-type electrochromic device, cycling stability

## Abstract

Five carbazole-containing polymeric membranes (PDTC, P(DTC-*co*-BTP), P(DTC-*co*-BTP2), P(DTC-*co*-TF), and P(DTC-*co*-TF2)) were electrodeposited on transparent conductive electrodes. P(DTC-*co*-BTP2) shows a high Δ*T* (68.4%) at 855 nm. The multichromic properties of P(DTC-*co*-TF2) membrane range between dark yellow, yellowish-green, gunmetal gray, and dark gray in various reduced and oxidized states. Polymer-based organic electrochromic devices are assembled using 2,2′-bithiophene- and 2-(2-thienyl)furan-based copolymers as anodic membranes, and poly(3,4-ethylenedioxythiophene)-poly(styrene sulfonic acid) (PEDOT-PSS) as the cathodic membrane. P(DTC-*co*-TF)/PEDOT-PSS electrochromic device (ECD) displays a high transmittance change (Δ*T*%) (43.4%) at 627 nm as well as a rapid switching time (less than 0.6 s) from a colored to a bleached state. Moreover, P(DTC-*co*-TF2)/PEDOT-PSS ECD shows satisfactory optical memory (the transmittance change is less than 2.9% in the colored state) and high coloration efficiency (512.6 cm^2^ C^−1^) at 627 nm.

## 1. Introduction 

Electrochromism refers to when electroactive species undergo a reversible change in optical absorption properties during the electrochemical oxidation/reduction process, and the species are electrochromic materials [1,2,3]. In the past 20 years, electrochromic materials and electrochemical membrane materials have been utilized in several fields such as for architectural windows, helmet visors, rearview mirrors, optical displays [4], fuel cells [5,6,7], and supercapacitors [8,9]. Electrochromic materials are generally classified into two categories: (a) inorganic complexes and transition metal oxides and (b) organic polymers and viologen derivatives [10]. Among these chromic materials, organic electrochromic polymers have gained extensive attention for promising applications in electrochromic electrodes owing to their short response time, facile color-tuning by chemical structures modification, low-power consumption, and good solution processability [11].

The most commonly used conjugated polymeric materials are polycarbazole [12,13], poly(3,4-ethylenedioxythiophene) (PEDOT) [14], polythiophene [15], polyfuran [16], polypyrrole [17], polyaniline [18], polytriphenylamine [19,20,21], and polyindole [22,23]. Polycarbazole and polytriphenylamine have been extensively applied for various electronic and optical devices owing to their high hole transporting mobility and good photoactive and electroactive performances [24]. Hsiao et al. published the electrochromic properties of carbazole- and triphenylamine-based polymers (P(PhCz-2Cz) and P(TPA-2Cz)). P(TPA-2Cz) film was colorless, dark green, and brown in its neutral, semi-oxidized, and fully oxidized states. P(PhCz-2Cz) displayed satisfactory electrochromic switching with a Δ*T*_max_ up to 35% at 1052 nm [25]. Oral et al. reported the electrochromic properties of a dual-type ECD, which employed polystyrene functionalized carbazole (PS-Carb) as the anodically coloring polymer. The Δ*T* of PS-Carb/PEDOT ECD was 38% at 640 nm and the response time was 1.1 s [26]. Polythiophene is a potentially useful electrode material due to its desirable stability to environment and high conductivity [27]. However, the onset oxidation potential of thiophene is higher than 1.5 V [28]. PEDOT is a derivative of polythiophene which introduces alkoxyl substituent groups into a thiophene ring. The onset oxidation potential of PEDOT is lower than polythiophene. PEDOT is light sky-blue and deep blue in doped and reduced states, respectively. Accordingly, PEDOT is a cathodically coloring layer in electrochromic devices (ECDs). Polyfuran possesses some particular properties such as high fluorescence, a high HOMO energy level, and adequate solubility [29]. Polyfurans synthesized using furan, bifuran, and trifuran have different electrochemical activities, polyfuran prepared using trifuran has the highest electrochemical activity [30].

In the present report, a homopolymer (PDTC), two 2,2′-bithiophene (BTP)-based copolymers (P(DTC-*co*-BTP) and P(DTC-*co*-BTP2)), and two 2-(2-thienyl) furan (TF)-based copolymers (P(DTC-*co*-TF) and P(DTC-*co*-TF2)) with different DTC/BTP and DTC/TF feed molar ratios are synthesized electrochemically to explore their promising applications in electrochromic products. A 3,6-di(2-thienyl)carbazole unit reveals that because two thiophenes are linked by a carbazole, the strong hole-transporting carbazole group raises the HOMO energy level of PDTC. Therefore, PDTC shows a lower onset potential of oxidation than that of polythiophene. The conjugated chain length of 2,2′-bithiophene is longer than that of thiophene. The onset oxidation potential of 2,2′-bithiophene is lower than that of thiophene. Accordingly, 2,2′-bithiophene is incorporated to the polymer backbone and electrochromic properties of 2,2′-bithiophene-based polymers are characterizated in this study. Moreover, 2-(2-thienyl)furan combines the properties of furan and thiophene, while the conjugated chain length of 2-(2-thienyl)furan is longer than those of furan and thiophene. It is interesting to explore the difference of electrochromic behaviors for 2,2′-bithiophene- and 2-(2-thienyl)furan-based polymer membranes. The electrode membranes could be useful for identifying additional transport properties to be associated to the electrochromic behavior. Furthermore, five ECDs consisted of PDTC, P(DTC-*co*-BTP), P(DTC-*co*-BTP2), P(DTC-*co*-TF), or P(DTC-*co*-TF2) as the anodic membrane, and PEDOT-PSS as the cathodic membrane were built and their spectroelectrochemical behaviors, electrochromic switching kinetics, and redox stability were explored in detail. 

## 2. Experimental

### 2.1. Materials

2,2′-bithiophene and 2-(2-thienyl)furan were purchased from Alfa Aesar and Sigma-Aldrich, respectively. 3,6-di(2-thienyl)carbazole (DTC) was synthesized according to previous procedures [31]. Electrolytes of ECDs were prepared using a mixture of poly(methyl methacrylate) (PMMA), propylene carbonate (PC), and LiClO_4_. The weight ratio of PMMA, PC, and LiClO_4_ is 33:53:14 [32].

### 2.2. Electrochemical Preparation of PDTC, P(DTC-co-BTP), P(DTC-co-BTP2), P(DTC-co-TF), P(DTC-co-TF2) Films

The electrosynthesis of PDTC, P(DTC-*co*-BTP), P(DTC-*co*-BTP2), P(DTC-*co*-TF), and P(DTC-*co*-TF2) membranes was implemented in a 0.2 M LiClO_4_/acetonitrile (ACN) solution, and the feed species and feed ratio of species for anodic polymers were displayed in Table 1. The 3,6-di(2-thienyl)carbazole- and thiophene derivatives-based homopolymer and copolymers were prepared potentiostatically at 1.2 V (vs. Ag/AgCl).

### 2.3. Assembly of Electrochromic Devices

Polymer ECDs were fabricated using PDTC, P(DTC-*co*-BTP), P(DTC-*co*-BTP2), P(DTC-*co*-TF), or P(DTC-*co*-TF2) film as the anodic electrochromic layer and PEDOT-PSS film as the cathodic electrochromic layer. The electrodeposited areas of polymer films were 1 × 1.5 cm^2^. The ECDs were assembled by arranging the anodic and cathodic films to face each other, and they were separated using a PMMA/PC/LiClO_4_ electrolyte.

### 2.4. Characterizations of Electrodes and Devices

The electrochemical properties of polymer films were characterized using a CHI627E electrochemical analyzer/workstation. The electrochemical experiments were operated using a three component cell. The working electrode, counter electrode, and reference electrode were an ITO coated glass plate, a platinum wire, and an Ag/AgCl electrode, respectively. The spectroelectrochemical experiments of polymer films and ECDs were measured using an electrochemical workstation and a JASCO V-630 UV-Visible spectrophotometer.

## 3. Results and Discussion

### 3.1. Electrochemical Characterization

**Figure 1:**
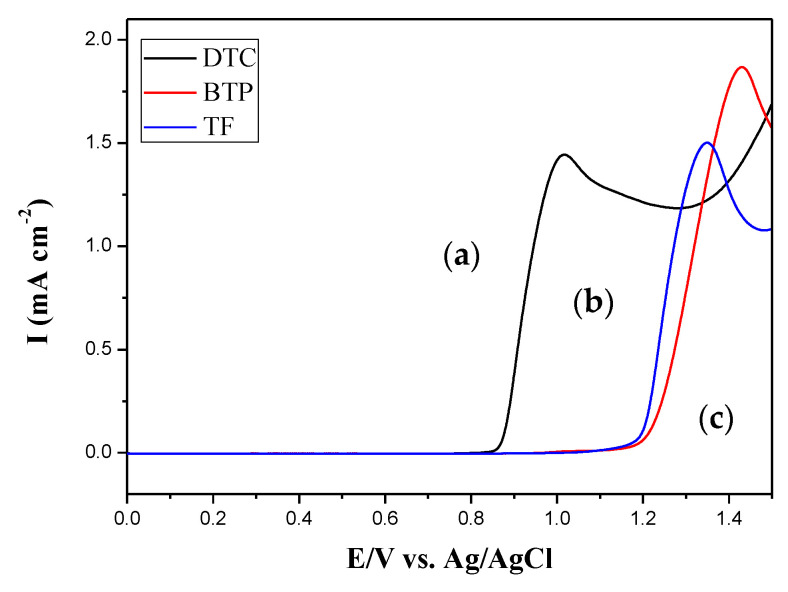
Electrooxidation curves of (**a**) 2 mM DTC, (**b**) 2 mM BTP, and (**c**) 2 mM TF in 0.2 M LiClO_4_/acetonitrile (ACN) at a scan rate of 100 mV s^−1^.

Figure 1 displays the electrooxidation curves of 2 mM DTC, 2 mM BTP, and 2 mM TF in 0.2 M LiClO_4_/ACN solution, indicating that the onset potentials (*E*_onset_) of oxidation for DTC, BTP, and TF were 0.88, 1.22, and 1.19 V, respectively. DTC displayed lower *E*_onset_ than those of BTP and TF, implying the electron donating carbazole unit of DTC decreased the *E*_onset_ significantly. Moreover, *E*_onset_ of 2-(2-thienyl)furan is slightly lower than that of 2,2′-bithiophene.

**Figure 2:**
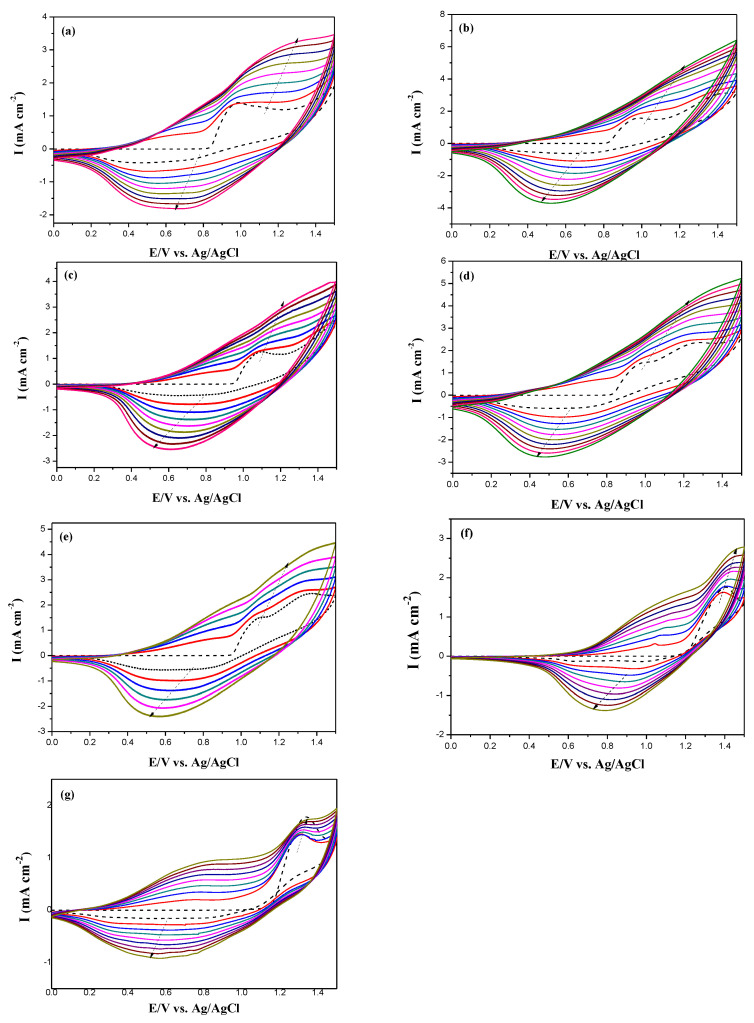
Electrochemical synthesis of (**a**) a homopolymer (PDTC), (**b**) P(DTC-*co*-BTP), (**c**) P(DTC-*co*-BTP2), (**d**) P(DTC-*co*-TF), (**e**) P(DTC-*co*-TF2), (**f**) PBTP, and (**g**) PTF in ACN solution at 100 mV s^−1^ on an ITO electrode.

Figure 2 shows the electrosynthesized curves of PDTC, P(DTC-*co*-BTP), P(DTC-*co*-BTP2), P(DTC-*co*-TF), P(DTC-*co*-TF2), PBTP, and PTF in 0.2 M LiClO_4_/ACN solution between 0.0 and 1.5 V. When the number of scanning curves increases, the peak current density increases with increasing number of cycles, indicating that the electrodeposition of polymer membranes is present on ITO glasses [33]. The oxidized and reduced peaks of five polymer films were quasi-reversible. The oxidation peaks of PDTC, P(DTC-*co*-BTP), P(DTC-*co*-BTP2), P(DTC-*co*-TF), P(DTC-*co*-TF2), PBTP, and PTF films were located at ca. 1.27, 1.2, 1.25, 1.23, 1.26, 1.37, and 1.31 V (vs. Ag/AgCl), respectively. The reduction peaks of PDTC, P(DTC-*co*-BTP), P(DTC-*co*-BTP2), P(DTC-*co*-TF), P(DTC-*co*-TF2), PBTP, and PTF were situated at ca. 0.65, 0.48, 0.52, 0.43, 0.50, 0.63, and 0.60 V (vs. Ag/AgCl), respectively. 

**Figure 3:**
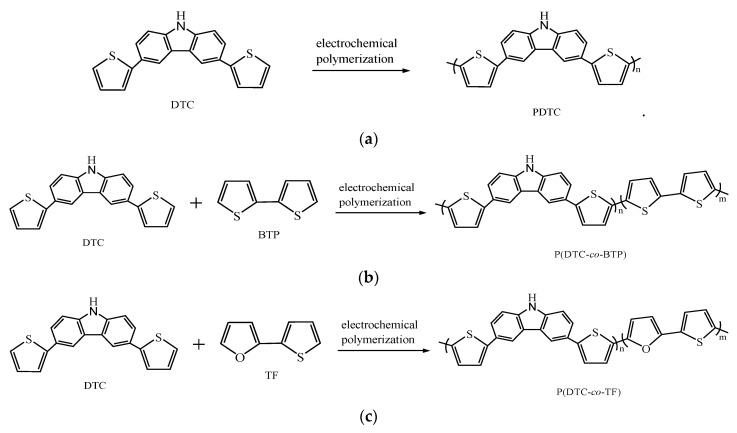
The electrochemical polymerization schemes of (**a**) PDTC, (**b**) P(DTC-*co*-BTP), and (**c**) P(DTC-*co*-TF).

The potentials and wave shapes of redox peaks of P(DTC-*co*-BTP), P(DTC-*co*-BTP2), P(DTC-*co*-TF), and P(DTC-*co*-TF2) are different to those of PDTC, PBTP, and PTF, proving the occurrence of copolymerization for P(DTC-*co*-BTP), P(DTC-*co*-BTP2), P(DTC-*co*-TF), and P(DTC-*co*-TF2) films. The general polymerization schemes of PDTC, P(DTC-*co*-BTP), and P(DTC-*co*-TF) are summarized in Figure 3. Specific capacitances of PDTC, P(DTC-*co*-BTP), P(DTC-*co*-BTP2), P(DTC-*co*-TF), and P(DTC-*co*-TF2) electrodes are 84, 109, 88, 98, and 92 F/g, respectively.

**Figure 4:**
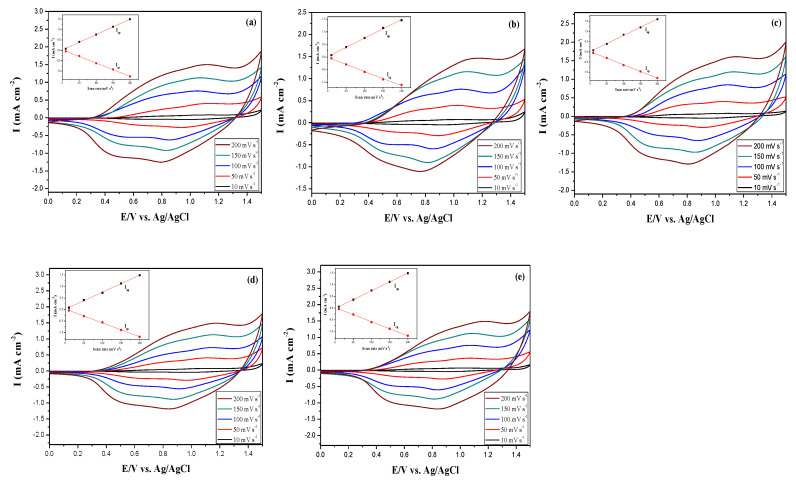
Cyclic voltammetry (CV) curves of (**a**) PDTC, (**b**) P(DTC-*co*-BTP), (**c**) P(DTC-*co*-BTP2), (**d**) P(DTC-*co*-TF), and (**e**) P(DTC-*co*-TF2) films at different scan rates between 10 and 200 mV s^−1^ in the LiClO_4_ + ACN solution. Inset: Plots of scan rate vs. anodic and cathodic peak current densities.

Figure 4 shows the CV curves of PDTC, P(DTC-*co*-BTP), P(DTC-*co*-BTP2), P(DTC-*co*-TF), and P(DTC-*co*-TF2) films at scan rates of 10, 50, 100, 150, and 200 mV s^−1^ in 0.2 M LiClO_4_/ACN. The anodic and cathodic peaks of PDTC, P(DTC-*co*-BTP), P(DTC-*co*-BTP2), P(DTC-*co*-TF), and P(DTC-*co*-TF2) membranes displayed *quasi-*reversible** behaviors and the inset in Figure 4 revealed that the redox peak current density increased with the increasing scan rate linearly, demonstrating that the oxidation and reduction *processes* of PDTC, P(DTC-*co*-BTP), P(DTC-*co*-BTP2), P(DTC-*co*-TF), and P(DTC-*co*-TF2) films were not *diffusion-*limited** [34].

### 3.2. Spectroelectrochemical Measurement of Polymers

**Figure 5:**
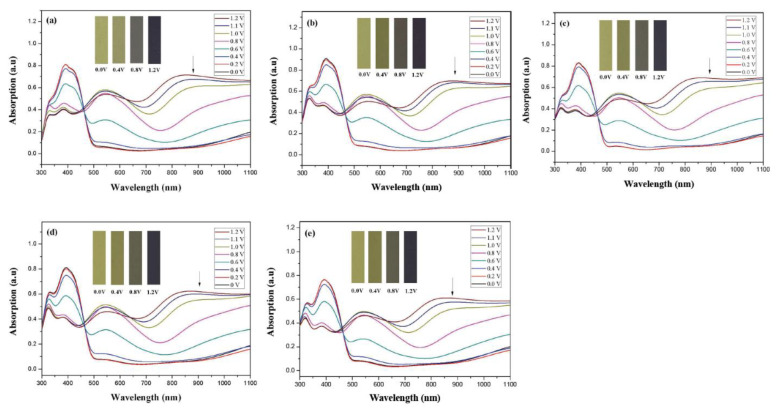
UV-Visible spectra of (**a**) PDTC, (**b**) P(DTC-*co*-BTP), (**c**) P(DTC-*co*-BTP2), (**d**) P(DTC-*co*-TF), and (**e**) P(DTC-*co*-TF2) in 0.2 M LiClO_4_/ACN solution.

Figure 5 displays absorption spectra of PDTC, P(DTC-*co*-BTP), P(DTC-*co*-BTP2), P(DTC-*co*-TF), and P(DTC-*co*-TF2) films in 0.2 M LiClO_4_/ACN. At 0.0 and 0.2 V, PDTC, P(DTC-*co*-BTP), P(DTC-*co*-BTP2), P(DTC-*co*-TF), and P(DTC-*co*-TF2) films display definite-transition bands at ca. 400 nm. After increasing the applied voltage gradually, new charge carrier bands appeared at a long wavelength region. The charge carrier bands of PDTC were located at 550 nm and 860 nm. For the corresponding copolymers, the latter charge carrier bands of P(DTC-*co*-BTP), P(DTC-*co*-BTP2), P(DTC-*co*-TF), and P(DTC-*co*-TF2) were situated at 875, 855, 870, and 855 nm, respectively.

PDTC displays four types of colors from neutral to oxidation state; the color of PDTC is light yellow at 0.0 V, mustard yellow at 0.4 V, gray at 0.8 V, and dark gray at 1.2 V. The 2,2′-bithiophene- and 2-(2-thienyl)furan-based copolymers show different electrochromic properties with PDTC homopolymer. The colors of P(DTC-*co*-BTP) electrode are light yellow, yellowish-green, gray, and dark gray at 0.0, 0.4, 0.8, and 1.2 V, respectively, whereas P(DTC-*co*-BTP2) is dark yellow, khaki, gray, and dark gray at 0.0, 0.4, 0.8 V, and 1.2 V, respectively. For 2-(2-thienyl)furan-based copolymers, P(DTC-*co*-TF) is mustard yellow, khaki, grey, and dark grey at 0.0, 0.4, 0.8, and 1.2 V, respectively, P(DTC-*co*-TF2) is dark yellow, yellowish-green, gunmetal gray, and dark gray at 0.0, 0.4, 0.8, and 1.2 V, respectively. The colorimetric values, CIE chromaticity values, and charts of PDTC, P(DTC-*co*-BTP), P(DTC-*co*-BTP2), P(DTC-*co*-TF), and P(DTC-*co*-TF2) at 0.0—1.0 V are summarized in Table 2. 

### 3.3. Electrochromic Switching of Anodic Polymers

**Figure 6:**
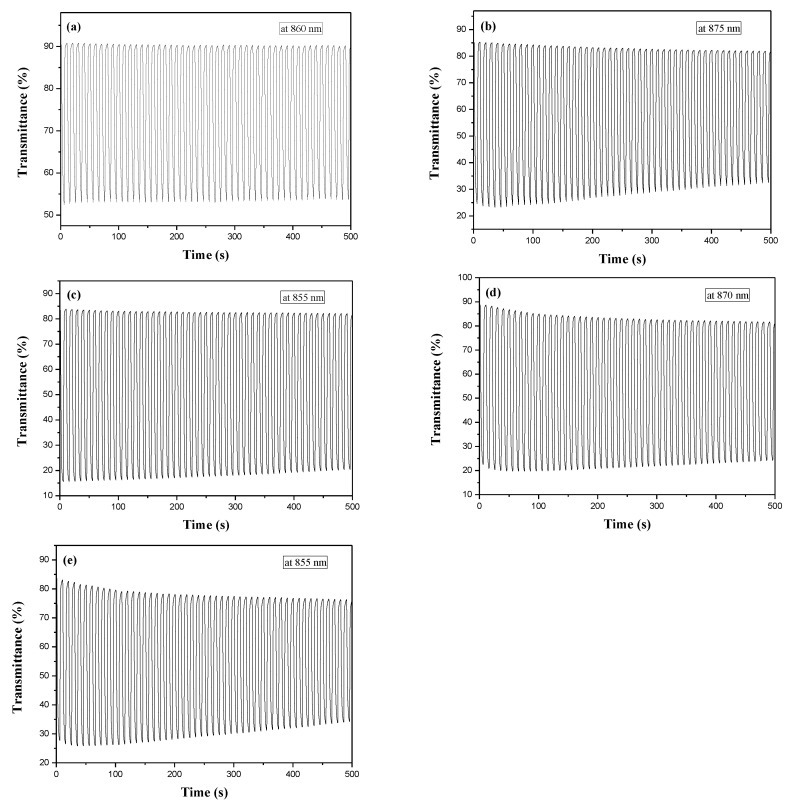
Optical contrast of (**a**) PDTC, (**b**) P(DTC-*co*-BTP), (**c**) P(DTC-*co*-BTP2), (**d**) P(DTC-*co*-TF), and (**e**) P(DTC-*co*-TF2) in 0.2 M LiClO_4_/ACN solution with a residence time of 5 s.

Figure 6 shows the transmittance-time plots of PDTC, P(DTC-*co*-BTP), P(DTC-*co*-BTP2), P(DTC-*co*-TF), and P(DTC-*co*-TF2) membranes caused by potential stepping between reduced and oxidized states. The residence time is 5 s. The Δ*T* of PDTC, P(DTC-*co*-BTP), P(DTC-*co*-BTP2), P(DTC-*co*-TF), and P(DTC-*co*-TF2) were 37.9% at 860 nm, 61.6% at 875 nm, 68.4% at 855 nm, 67.3% at 870 nm, and 56.1% at 855 nm, respectively (Table 3). The Δ*T* of 2,2′-bithiophene- and 2-(2-thienyl)furan-based copolymers (P(DTC-*co*-BTP), P(DTC-*co*-BTP2), P(DTC-*co*-TF), and P(DTC-*co*-TF2)) were higher than that of PDTC in 0.2 M LiClO_4_/ACN, inferring that copolymers containing 2,2′-bithiophene or 2-(2-thienyl)furan groups increased Δ*T* significantly. Moreover, the Δ*T* of P(DTC-*co*-BTP2) was higher than those reported for P(PtCz-*co*-BTP2) [35], PITID-2 [36], P(HoT-BSe-OF) [37], PDTCZ-2 [38], and PI-6D [39] (Table 4). The bleaching response time (*τ*_b_) and coloring response time (*τ*_c_) needed to reach 90% of the maximum transmittance change were determined to be 0.9–3.1 s for these polymers. The coloration efficiency (*η*) is a significant factor for effective utilization of polymers in electrochromic devices and it can be obtained using the equation below [40]:

(1) $$\eta =\;\frac{\Delta \mathrm{OD}}{Q_{d}}$$

where ΔOD and *Q*_d_ represent the change of optical density and the injected charges divided by active electrode area, respectively. As listed in Table 3, the *η* of PDTC, P(DTC-*co*-BTP), P(DTC-*co*-BTP2), P(DTC-*co*-TF), and P(DTC-*co*-TF2) were 125.8 cm^2^ C^−1^ at 860 nm, 141.5 cm^2^ C^−1^ at 875 nm, 159.4 cm^2^ C^−1^ at 855 nm, 161.6 cm^2^ C^−1^ at 870 nm, and 152.9 cm^2^ C^−1^ at 855 nm, respectively. P(DTC-*co*-BTP2) shows higher *η* than that of P(HoT-BSe-OF) [37]. However, P(DTC-*co*-BTP2) shows a lower *η* than those of PITID-2 [36], PDTCZ-2 [38], and PI-6D [39] (Table 4).

### 3.4. Spectroelectrochemical Properties of ECDs

**Figure 7:**
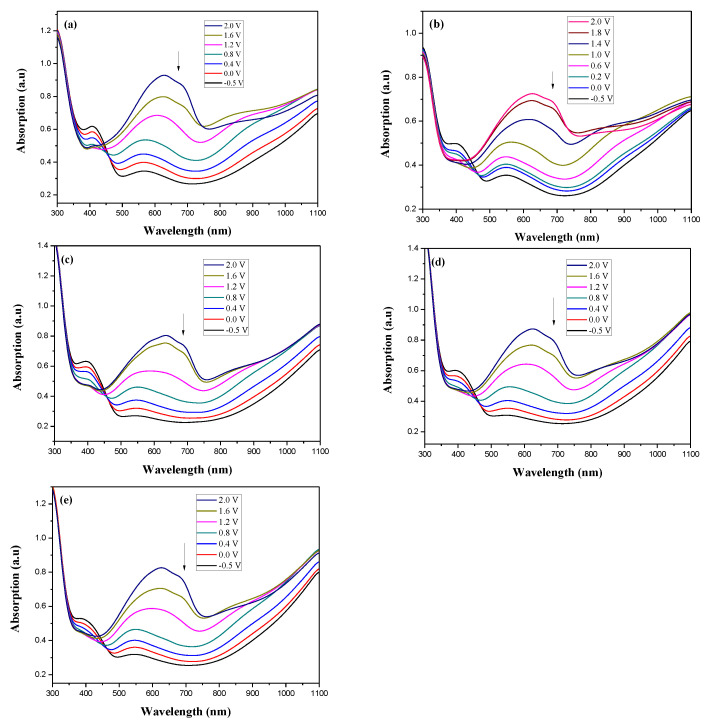
UV-Visible spectra of (**a**) PDTC/PEDOT-PSS, (**b**) P(DTC-*co*-BTP)/PEDOT-PSS, (**c**) P(DTC-*co*-BTP2)/PEDOT-PSS, (**d**) P(DTC-*co*-TF)/PEDOT-PSS, and (**e**) P(DTC-*co*-TF2)/PEDOT-PSS ECDs.

Figure 7 showed the absorption spectra of PDTC/PEDOT-PSS, P(DTC-*co*-BTP)/PEDOT-PSS, P(DTC-*co*-BTP2)/PEDOT-PSS, P(DTC-*co*-TF)/PEDOT-PSS, and P(DTC-*co*-TF2)/PEDOT-PSS ECDs at assorted potentials. The five ECDs showed distinct UV-Visible bands *at* ca. 400 nm at around −0.5 V, which could be ascribed to the UV-Visible bands of PDTC, P(DTC-*co*-BTP), P(DTC-*co*-BTP2), P(DTC-*co*-TF), and P(DTC-*co*-TF2) at low potential zone. The UV-Visible bands of PDTC, P(DTC-*co*-BTP), P(DTC-*co*-BTP2), P(DTC-*co*-TF), and P(DTC-*co*-TF2) bleached with increasing potentials, and new visible peaks appeared bit by bit at 627–630 nm. This could be assigned to the reduction of PEDOT-PSS bit by bit. The colors of PDTC/PEDOT-PSS ECD are gray, dark gray, and *navy blue* at −0.5, 0.8, and 2.0 V, respectively. P(DTC-*co*-BTP)/PEDOT-PSS ECD is bright gray, dark gray, and deep blue at −0.5, 0.6, and 2.0 V, respectively. P(DTC-*co*-BTP2)/PEDOT-PSS ECD is bright gray, gunmetal grey, and berlin blue at −0.5, 0.8, and 2.0 V, respectively. P(DTC-*co*-TF)/PEDOT-PSS ECD is bright gray, iron grey, and sapphire at −0.5, 0.8, and 2.0 V, respectively. P(DTC-*co*-TF2)/PEDOT-PSS ECD is bright gray, gunmetal grey, and berlin blue at −0.5, 0.8, and 2.0 V, respectively. The photos, L*, a*, b*, *x*, *y*, and CIE diagrams of PDTC/PEDOT-PSS, P(DTC-*co*-BTP)/PEDOT-PSS, P(DTC-*co*-BTP2)/PEDOT-PSS, P(DTC-*co*-TF)/PEDOT-PSS, and P(DTC-*co*-TF2)/PEDOT-PSS ECDs at different potentials are summarized in Table 5.

### 3.5. Colorless-to-Colorful Switching of ECDs

**Figure 8:**
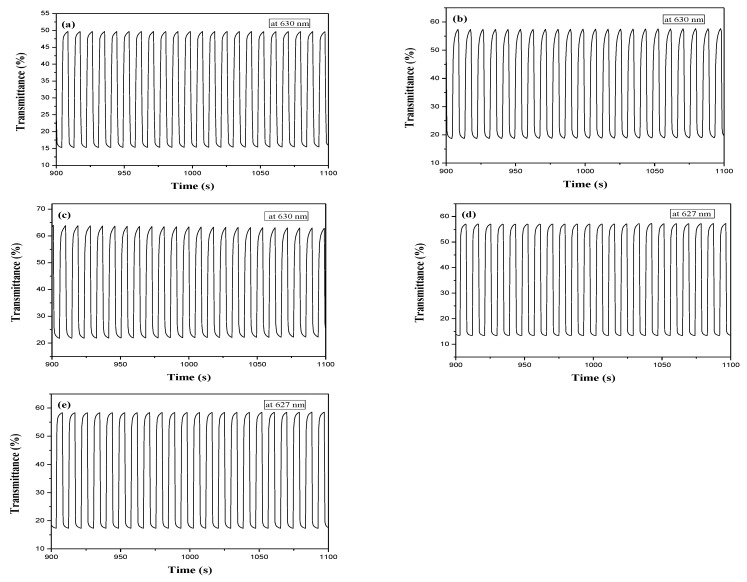
Optical contrast of (**a**) PDTC/PEDOT-PSS, (**b**) P(DTC-*co*-BTP)/PEDOT-PSS, (**c**) P(DTC-*co*-BTP2)/PEDOT-PSS, (**d**) P(DTC-*co*-TF)/PEDOT-PSS, and (**e**) P(DTC-*co*-TF2)/PEDOT-PSS ECDs with a residence time of 5 s.

Figure 8 showed the potential stepping of PDTC/PEDOT-PSS, P(DTC-*co*-BTP)/PEDOT-PSS, P(DTC-*co*-BTP2)/PEDOT-PSS, P(DTC-*co*-TF)/PEDOT-PSS, and P(DTC-*co*-TF2)/PEDOT-PSS ECDs between colorless and colorful states with a residence time of 5 s. The ΔOD, Δ*T*, *τ*_b_, and *τ*_c_ of PDTC/PEDOT-PSS, P(DTC-*co*-BTP)/PEDOT-PSS, P(DTC-*co*-BTP2)/PEDOT-PSS, P(DTC-*co*-TF)/PEDOT-PSS, and P(DTC-*co*-TF2)/PEDOT-PSS ECDs are displayed in Table 6. The Δ*T*_max_ of PDTC/PEDOT-PSS, P(DTC-*co*-BTP)/PEDOT-PSS, P(DTC-*co*-BTP2)/PEDOT-PSS, P(DTC-*co*-TF)/PEDOT-PSS, and P(DTC-*co*-TF2)/PEDOT-PSS ECDs are 34.3% at 630 nm, 38.7% at 630 nm, 41.6% at 630 nm, 43.4% at 627 nm, and 41.1% at 627 nm, respectively. P(DTC-*co*-TF)/PEDOT-PSS ECD showed the highest Δ*T*_max_, and P(DTC-*co*-BTP)/PEDOT-PSS, P(DTC-*co*-BTP2)/PEDOT-PSS, P(DTC-*co*-TF)/PEDOT-PSS, and P(DTC-*co*-TF2)/PEDOT-PSS ECDs showed higher Δ*T*_max_ than that of PDTC/PEDOT-PSS ECD. This implies that the employment of copolymers (P(DTC-*co*-BTP), P(DTC-*co*-BTP2), P(DTC-*co*-TF), and P(DTC-*co*-TF2)) as the anodic layers results in a higher Δ*T*_max_ at ca. 627 nm than that of homopolymers (PDTCs). Table 4 lists Δ*T*’s comparisons of P(DTC-*co*-TF)/PEDOT-PSS ECD with reported ECDs, showing that P(DTC-*co*-2BTP)/PEDOT-PSS ECD displays a higher Δ*T* than those reported for PETI/PEDOT [41], P(PS-Carb)/PEDOT [26], P(BCO)/PEDOT [42], and P(DiCP-*co*-CPDTK)/PEDOT-PSS ECDs [43]. 

The *τ*_b_ and *τ*_c_ of PDTC/PEDOT-PSS, P(DTC-*co*-BTP)/PEDOT-PSS, P(DTC-*co*-BTP2)/PEDOT-PSS, P(DTC-*co*-TF)/PEDOT-PSS, and P(DTC-*co*-TF2)/PEDOT-PSS ECDs in Table 6 are in the range of 0.3–0.9 s. *τ*_b_ and *τ*_c_ of five ECDs were shorter than their corresponding anodes in 0.2 M LiClO_4_/ACN, revealing the ECDs switched color quicker than the anodes in 0.2 M LiClO_4_/ACN from the colored to the bleached state [44].

The *η* of PDTC/PEDOT-PSS, P(DTC-*co*-BTP)/PEDOT-PSS, P(DTC-*co*-BTP2)/PEDOT-PSS, P(DTC-*co*-TF)/PEDOT-PSS, and P(DTC-*co*-TF2)/PEDOT-PSS ECDs are 420.7 cm^2^ C^−1^ at 630 nm, 537.4 cm^2^ C^−1^ at 630 nm, 398.3 cm^2^ C^−1^ at 630 nm, 496.0 cm^2^ C^−1^ at 627 nm, and 512.6 cm^2^ C^−1^ at 627 nm, respectively. Among these ECDs, P(DTC-*co*-BTP) with a feed molar ratio of DTC/BTP = 1/1 displays the highest *η*. Table 4 displays *η*’s comparisons of P(DTC-*co*-TF)/PEDOT-PSS ECD with reported ECDs, while P(DTC-*co*-TF)/PEDOT-PSS ECD shows a greater *η* than that reported for PETI/PEDOT ECD [41]. However, P(DTC-*co*-TF)/PEDOT-PSS ECD displays a lower *η* than that reported for P(DiCP-*co*-CPDTK)/PEDOT-PSS ECD [43].

### 3.6. Optical Memory Influences of ECDs

**Figure 9:**
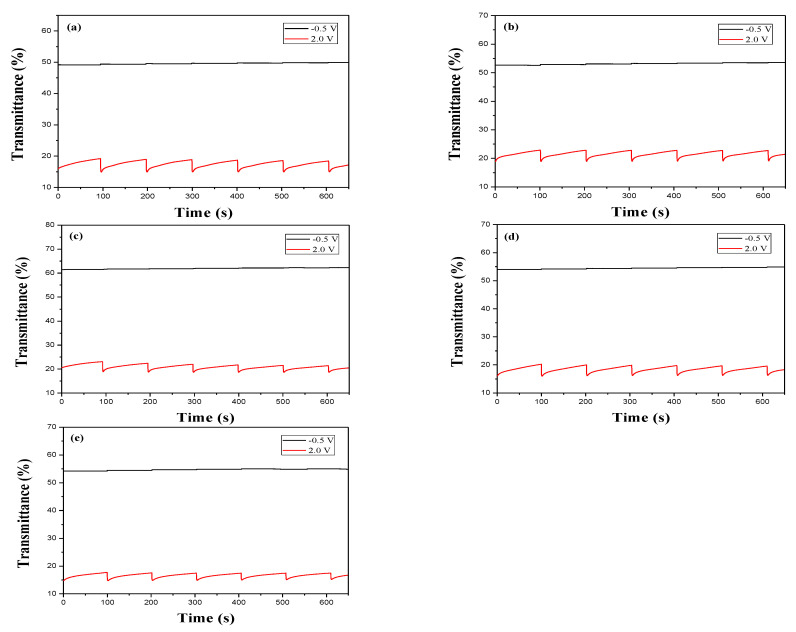
Open circuit stability of (**a**) PDTC/PEDOT-PSS, (**b**) P(DTC-*co*-BTP)/PEDOT-PSS, (**c**) P(DTC-*co*-BTP2)/PEDOT-PSS, (**d**) P(DTC-*co*-TF)/PEDOT-PSS, and (**e**) P(DTC-*co*-TF2)/PEDOT-PSS ECDs.

The optical memory influences of PDTC/PEDOT-PSS, P(DTC-co-BTP)/PEDOT-PSS, P(DTC-co-BTP2)/PEDOT-PSS, P(DTC-co-TF)/PEDOT-PSS, and P(DTC-co-TF2)/PEDOT-PSS ECDs were monitored in the colored and bleached states by exerting the voltage for 1 sec for each 100 sec interval. Figure 9 displayed that the transmittances of PDTC/PEDOT-PSS, P(DTC-co-BTP)/PEDOT-PSS, P(DTC-co-BTP2)/PEDOT-PSS, P(DTC-co-TF)/PEDOT-PSS, and P(DTC-co-TF2)/PEDOT-PSS ECDs experienced almost no change in the bleached state. On the other hand, the transmittance changes of PDTC/PEDOT-PSS, P(DTC-co-BTP)/PEDOT-PSS, P(DTC-co-BTP2)/PEDOT-PSS, P(DTC-co-TF)/PEDOT-PSS, and P(DTC-co-TF2)/PEDOT-PSS ECDs in the colored state were less electrochemically stable than those in the bleached state. However, the change of transmittance for ECDs in the colored states was less than 4.3%. P(DTC-co-TF2)/PEDOT-PSS ECD showed the lowest transmittance change (2.9%) in the colored state and P(DTC-co-BTP2)/PEDOT-PSS ECD showed the lowest transmittance change (0.2%) in the bleached state. Considering the aforementioned results, P(DTC-co-BTP)/PEDOT-PSS, P(DTC-co-BTP2)/PEDOT-PSS, P(DTC-co-TF)/PEDOT-PSS, and P(DTC-co-TF2)/PEDOT-PSS ECDs display acceptable optical memory.

### 3.7. Redox Stability of ECDs

**Figure 10:**
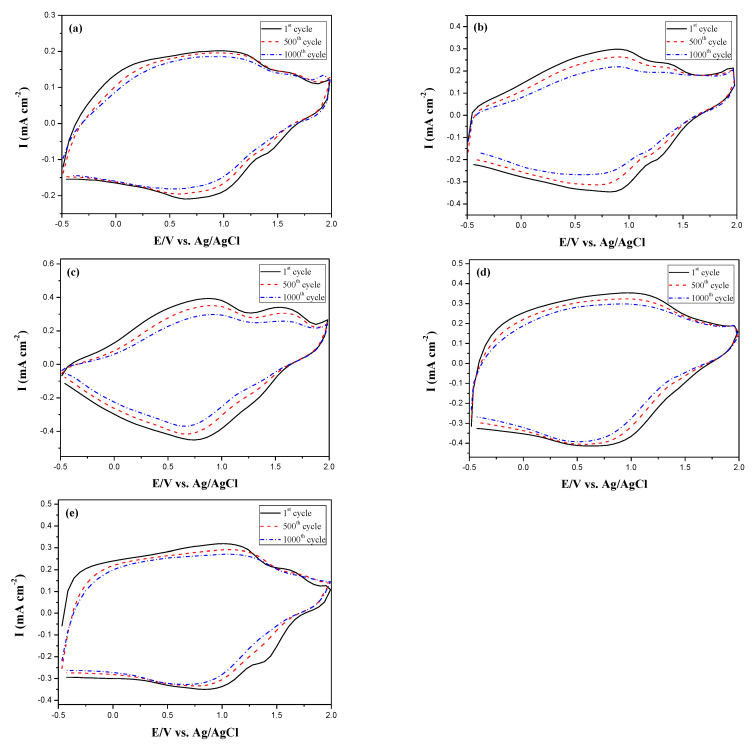
Cyclic voltammograms of (**a**) PDTC/PEDOT-PSS, (**b**) P(DTC-*co*-BTP)/PEDOT-PSS, (**c**) P(DTC-*co*-BTP2)/PEDOT-PSS, (**d**) P(DTC-*co*-TF)/PEDOT-PSS, and (**e**) P(DTC-*co*-TF2)/PEDOT-PSS devices with a scan rate of 500 mV s^−1^ between the 1st and 1000th cycles.

The long-term cycling stability measurement of PDTC/PEDOT-PSS, P(DTC-*co*-BTP)/PEDOT-PSS, P(DTC-*co*-BTP2)/PEDOT-PSS, P(DTC-*co*-TF)/PEDOT-PSS, and P(DTC-*co*-TF2)/PEDOT-PSS ECDs was carried out using CV at the first, 500th and 1000th cycles [45]. From the inspection of redox ability in Figure 10, 90.3%, 87.6%, 85.1%, 91.5%, and 89.7% of electroactivity are conserved at the 500th cycle, and 86.7%, 74.4%, 72.1%, 83.7%, and 84.6% of electroactivity are conserved at the 1000th cycle for PDTC/PEDOT-PSS, P(DTC-*co*-BTP)/PEDOT-PSS, P(DTC-*co*-BTP2)/PEDOT-PSS, P(DTC-*co*-TF)/PEDOT-PSS, and P(DTC-*co*-TF2)/PEDOT-PSS ECDs, respectively. P(DTC-*co*-TF)/PEDOT-PSS and P(DTC-*co*-TF2)/PEDOT-PSS ECDs showed better cycling stability than those of P(DTC-*co*-BTP)/PEDOT-PSS and P(DTC-*co*-BTP2)/PEDOT-PSS ECDs, displaying ECDs employed 2-(2-thienyl)furan-containing P(DTC-*co*-TF) (or P(DTC-*co*-TF2)) as anodic layer led to a better cycling stability than that of 2,2′-bithiophene-containing P(DTC-*co*-BTP) (or P(DTC-*co*-BTP2)).

## 4. Conclusions

A series of ECDs’ anodic materials (PDTC, P(DTC-*co*-BTP), P(DTC-*co*-BTP2), P(DTC-*co*-TF), and P(DTC-*co*-TF2)) were prepared electrochemically. The 2,2′-bithiophene- and 2-(2-thienyl)furan-based copolymers prepared with various monomer feed ratios displayed various colorimetry, spectroelectrochemical, and electrochromic switching performances. The colors of P(DTC-*co*-BTP) electrode are light yellow, yellowish-green, gray, and dark gray at 0.0, 0.4, 0.8, and 1.2 V, respectively. The Δ*T* of P(DTC-*co*-BTP), P(DTC-*co*-BTP2), P(DTC-*co*-TF), and P(DTC-*co*-TF2) in solutions were 61.6% at 875 nm, 68.4% at 855 nm, 67.3% at 870 nm, and 56.1% at 855 nm, respectively. Dual type polymer ECDs which consisted of 2,2′-bithiophene- and 2-(2-thienyl)furan-based anodically coloring membranes and a PEDOT-PSS cathodically coloring membrane were made. The transmittance changes of ECDs’ optical memory test in the colored and bleached states were less than 4.3% and 0.4%, respectively. P(DTC-*co*-TF)/PEDOT-PSS ECD displays the highest Δ*T* (43.4% at 627 nm), whereas P(DTC-*co*-BTP)/PEDOT-PSS ECD shows the highest *η* (537.4 cm^2^ C^−1^ at 630 nm). In view of the aforementioned performances, 2,2′-bithiophene- and 2-(2-thienyl)furan-based electrochromic membranes are suitable to apply in electrochromic goggles, e-skins, textiles, and wearable display devices.

## Figures and Tables

**Table 1:** Feed species of anodic polymers.

Electrodes	Anodic Polymers	Feed Species of Anodic Polymers	Feed Molar Ratio of Anodic Polymers
(a)	PDTC	2 mM DTC	Neat PDTC
(b)	P(DTC-*co*-BTP)	2 mM DTC + 2 mM BTP	DTC:BTP = 1:1
(c)	P(DTC-*co*-BTP2)	2 mM DTC + 4 mM BTP	DTC:BTP = 1:2
(d)	P(DTC-*co*-TF)	2 mM DTC + 2 mM TF	DTC:TF = 1:1
(e)	P(DTC-*co*-TF2)	2 mM DTC + 4 mM TF	DTC:TF = 1:2

**Table 2:** Colorimetric values (L*, a*, and b*), CIE chromaticity values (*x*, *y*), and CIE diagrams of (a) PDTC, (b) P(DTC-*co*-BTP), (c) P(DTC-*co*-BTP2), (d) P(DTC-*co*-TF), and (e) P(DTC-*co*-TF2) at various applied potentials.

Films	Potential (V)	L*	a*	b*	*x*	*y*	Diagrams
(a)	0.0	70.45	−3.23	7.29	0.3235	0.3493	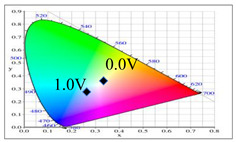
	0.4	67.3	−1.07	2.08	0.3157	0.3351	
	0.6	62.34	1.36	−5.18	0.3024	0.3144	
	0.8	55.89	−0.11	−13.45	0.2766	0.2913	
	1.0	52.01	−1.19	−17.14	0.2624	0.2794	
(b)	0.0	75.43	−2.85	11.33	0.3321	0.3571	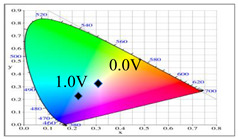
	0.4	72.02	−0.36	5.82	0.325	0.3429	
	0.6	66.68	2.7	−2.94	0.3106	0.3195	
	0.8	60.53	2.92	−12.11	0.2876	0.2947	
	1.0	54.06	1.08	−19.74	0.2614	0.2721	
(c)	0.0	95.82	−13.98	44.81	0.3683	0.4233	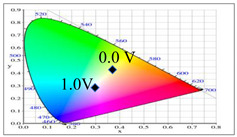
	0.4	92.85	−11.67	40.39	0.366	0.4154	
	0.6	78.43	−1.46	14.04	0.3393	0.3609	
	0.8	65.19	10.53	−12.6	0.3012	0.2899	
	1.0	62.19	11.62	−15.4	0.2957	0.2810	
(d)	0.0	93.35	−12.65	41.74	0.3665	0.4184	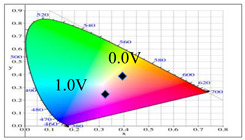
	0.4	90.01	−9.75	35.87	0.3627	0.4071	
	0.6	76.91	0.4	11.45	0.3375	0.354	
	0.8	65.57	10.39	−10.81	0.3053	0.2944	
	1.0	64.01	9.85	−11.98	0.3013	0.2914	
(e)	0.0	93.22	−11.1	39.37	0.3651	0.4126	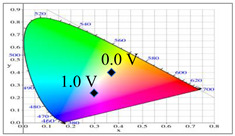
	0.4	90.72	−8.62	34.39	0.3616	0.4027	
	0.6	80.06	−0.69	14.72	0.3415	0.3611	
	0.8	67.38	10.15	−10.48	0.3059	0.296	
	1.0	65.00	10.16	−12.55	0.3006	0.2902	

**Table 3:** Optical properties of electrodes.

Electrodes	λ (nm)	*T*_ox_	*T*_red_	Δ*T*	ΔOD	*Q_d_* (mC cm^−2^)	*η* (cm^2^ C^−1^)	*τ*_c_ (s)	*τ*_b_ (s)
PDTC	860	52.8	90.7	37.9	0.24	1.87	125.8	3.1	1.2
	550	31.9	68.4	36.5	0.33	1.85	178.4	0.9	1.9
P(DTC-*co*-BTP)	875	23.6	85.2	61.6	0.56	3.94	141.5	3.1	1
	550	21.9	62.1	40.2	0.45	3.41	131.9	1.4	1.7
P(DTC-*co*-BTP2)	855	15.5	83.9	68.4	0.73	4.6	159.4	1.9	1
	550	23.7	65.6	41.9	0.44	3.36	130.9	1.3	1.9
P(DTC-*co*-TF)	870	21.3	88.6	67.3	0.62	3.83	161.6	3.4	1.8
	550	25.8	59.8	34	0.36	3.65	98.6	1.3	1.8
P(DTC-*co*-TF2)	855	26.8	82.9	56.1	0.49	3.21	152.9	3.1	1.2
	550	27.2	57.9	30.7	0.33	3.7	89.2	1.4	1.6

**Table 4:** Transmittance changes and coloration efficiencies of polymer films and ECDs.

Polymer Films or ECD Configurations	*λ* (nm)	Δ*T* (%)	*η* (cm^2^ C^−1^)	References
P(PtCz-*co*-BTP2)	565	34	-	[35]
PITID-2	675	18	172	[36]
P(HoT-BSe-OF)	860	29	142	[37]
PDTCZ-2	898	30.7	169	[38]
PI-6D	568	57	250	[39]
P(DTC-*co*-BTP2)	855	68.4	159.4	This work
PETI/PEDOT	600	32	290	[41]
P(PS-Carb)/PEDOT	640	38	-	[26]
P(BCO)/PEDOT	620	35	-	[42]
P(DiCP-*co*-CPDTK)/PEDOT-PSS	635	38.2	633.8	[43]
P(DTC-*co*-TF)/PEDOT-PSS	627	43.4	496.0	This work

**Table 5:** Electrochromic photographs, colorimetric values (L*, a*, and b*) and CIE chromaticity values (*x*, *y*) of (a) PDTC/PEDOT-PSS, (b) P(DTC-*co*-BTP)/PEDOT-PSS, (c) P(DTC-*co*-BTP2)/PEDOT-PSS, (d) P(DTC-*co*-TF)/PEDOT-PSS, and (e) P(DTC-*co*-TF2)/PEDOT-PSS ECDs at various potentials.

ECDs	Potential (V)	Photographs	L*	a*	b*	*x*	*y*	Diagrams
(a)	−0.5	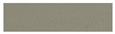	73.76	−5.48	13.00	0.3314	0.3640	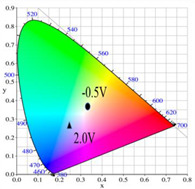
	0.0	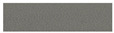	70.45	−3.23	7.29	0.3235	0.3493	
	0.8	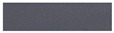	62.34	1.36	−5.18	0.3024	0.3144	
	1.2	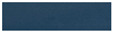	55.89	−0.11	−13.45	0.2766	0.2913	
	1.6	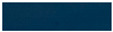	52.01	−1.19	−17.14	0.2624	0.2794	
	2.0	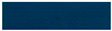	48.40	−2.02	−21.74	0.2453	0.2638	
(b)	−0.5	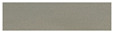	73.29	−0.24	5.87	0.3251	0.3427	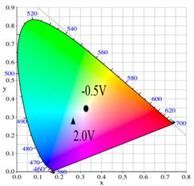
	0.0	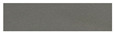	70.17	2.45	−0.86	0.3180	0.3302	
	0.6	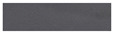	68.05	3.90	−4.33	0.3095	0.3154	
	1.4	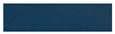	59.39	1.73	−14.33	0.2795	0.2894	
	1.8	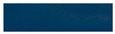	56.12	1.21	−17.49	0.2688	0.2795	
	2.0	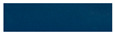	55.16	0.83	−18.71	0.2643	0.2759	
(c)	−0.5	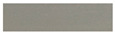	78.63	−5.24	16.98	0.3390	0.3708	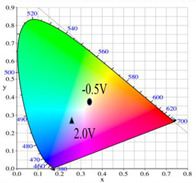
	0.0	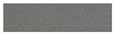	75.43	−2.85	11.33	0.3321	0.3571	
	0.8	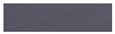	66.68	2.70	−2.94	0.3106	0.3195	
	1.2	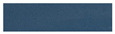	60.53	2.92	−12.11	0.2876	0.2947	
	1.6	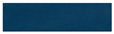	54.06	1.08	−19.74	0.2614	0.2721	
	2.0	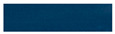	52.64	0.45	−21.13	0.2555	0.2676	
(d)	−0.5	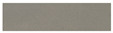	75.91	−4.19	14.22	0.3358	0.3647	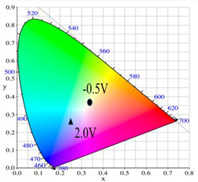
	0.0	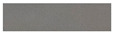	73.10	−1.97	8.57	0.3281	0.3506	
	0.8	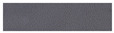	64.62	3.02	−5.16	0.3058	0.3135	
	1.2	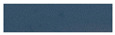	57.52	1.82	−14.93	0.2772	0.2867	
	1.6	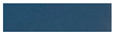	52.82	1.59	−19.79	0.2614	0.2706	
	2.0	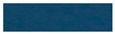	49.75	0.79	−23.37	0.2477	0.2585	
(e)	−0.5	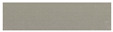	73.57	−2.28	9.72	0.3300	0.3534	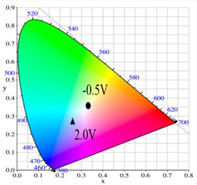
	0.0	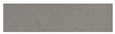	71.28	−0.60	4.92	0.3227	0.3411	
	0.8	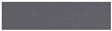	64.12	3.47	−6.6	0.3030	0.3094	
	1.2	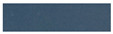	58.17	2.20	−14.48	0.2794	0.288	
	1.6	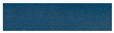	54.37	1.44	−18.42	0.2657	0.2756	
	2.0	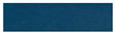	51.95	0.55	−20.23	0.2575	0.2694	

**Table 6:** Optical properties of devices.

ECDs	*T*_ox_	*T*_red_	Δ*T*	ΔOD	*Q_d_* (mC cm^−2^)	*η* (cm^2^∙C^−1^)	τ_c/_s	τ_b/_s
PDTC/PEDOT-PSS (630 nm)	15.4	49.7	34.3	0.509	1.21	420.7	0.9	0.7
P(DTC-*co*-BTP)/PEDOT-PSS (630 nm)	18.6	57.3	38.7	0.489	0.91	537.4	0.6	0.4
P(DTC-*co*-BTP2)/PEDOT-PSS (630 nm)	21.9	63.5	41.6	0.462	1.16	398.3	0.9	0.5
P(DTC-*co*-TF)/PEDOT-PSS (627 nm)	13.5	56.9	43.4	0.625	1.26	496	0.6	0.3
P(DTC-*co*-TF2)/PEDOT-PSS (627 nm)	17.3	58.4	41.1	0.528	1.03	512.6	0.5	0.4
